# Optimized W1/O/W2 Double Emulsions for *Lactobacillus paracasei* Encapsulation: Stability, Viability, and Anti‐Inflammatory Efficacy in a Rat Model of Ulcerative Colitis

**DOI:** 10.1002/fsn3.72598

**Published:** 2026-11-11

**Authors:** Leila Mahmoudzadeh, Sara Chadorshabi, Ehsan Shamloo, Mahnaz Tabibiazar, Aziz Homayouni‐Rad, Maryam Mahmoudzadeh, Hashem Andishmand

**Affiliations:** ^1^ Division of Immunology, Department of Microbiology, Faculty of Veterinary Medicine Urmia University Urmia Iran; ^2^ Student Research Committee, Department of Food Science and Technology, Faculty of Nutrition and Food Science Tabriz University of Medical Sciences Tabriz Iran; ^3^ Department of Food Science and Technology, Faculty of Nutrition and Food Science Tabriz University of Medical Sciences Tabriz Iran; ^4^ Nutrition Health Research Center, Institute of Health Sciences and Technology Hamadan University of Medical Sciences Hamadan Iran; ^5^ Research Center for Food Hygiene and Safety, School of Public Health Shahid Sadoughi University of Medical Sciences Yazd Iran; ^6^ Department of Food Hygiene and Safety, School of Public Health Shahid Sadoughi University of Medical Sciences Yazd Iran

**Keywords:** double emulsion, encapsulation efficiency, rheological properties, ulcerative colitis

## Abstract

In this work, the optimization of water‐in‐oil‐in‐water (W1/O/W2) emulsions for the encapsulation of *Lactobacillus paracasei* (CRL 431) was investigated to improve probiotic protection, delivery, and therapeutic performance in the treatment of ulcerative colitis (UC). The contents of ethyl cellulose (EC) in the oil phase and guar gum (GG) in the inner and outer aqueous phases were tuned using a Box–Behnken design (BBD), in which encapsulation efficiency, emulsion stability, and particle size were used as response variables to identify the optimal formulation. The optimal concentration (OC), including 0.25% GG in the inner phase, 0.74% GG in the outer phase, and 2.8% EC, attained an encapsulation efficiency of 95.8% and emulsion stability of 90.5%. The in vitro assessment of acid and bile tolerance for encapsulated bacteria in the OC formulation and the formulation stabilized with the highest concentrations (HC) of EC and GG revealed no significant differences. Rheological analysis showed that the OC formulation had lower viscosity and a less gel‐like, weakly structured behavior than the HC formulation. The OC formulation significantly reduced DAI and improved mucosal architecture in a Wistar rat model of UC. This effect was stronger than that of the HC formulation, indicating that this formulation can be used as a functional food in the alleviation of UC effects. The higher performance of the OC emulsion is attributed to its superior physical properties, which contribute to the stability, viability, and gastrointestinal delivery potential of *L. paracasei* . These findings illustrate the efficacy of tailored W1/O/W2 emulsions as a delivery mechanism for probiotics in the management of UC. Future clinical trials are advised to assess its relevance to human health.

## Graphical abstract

*Lactobacillus paracasei* delivery by double emulsion enhances survival and reduces experimental colitis.

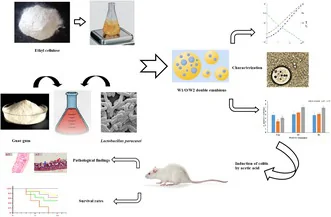

## 1 Introduction

*Lactobacillus paracasei* subsp. *paracasei* 431 belongs to lactic acid bacteria and exhibits probiotic characteristics. Living microorganisms that improve a host's health when ingested in greater quantities (9 log CFU/mL) are referred to as “probiotics” (Anal and Singh 2007; Norouzi et al. 2019). Nevertheless, probiotics are sensitive to processing, storage, and gastrointestinal conditions; maintaining their viability remains a major challenge. Consequently, encapsulation strategies that create physical barriers around cells have received significant attention (Burgain et al. 2011). To increase bacterial resistance to harsh environments, various encapsulation methods have been developed, including emulsification, coacervation, freeze‐drying, spray‐drying, extrusion, and electrospraying (Misra et al. 2021; Rabbani et al. 2021). Beneficial protective effects and improved bacterial viability in food matrices such as synbiotic bread—defined as a mixture comprising live microorganisms and substrate(s) selectively utilized by host microorganisms that confers a health benefit on the host (Swanson et al. 2020)—have been observed with alginate‐based systems, sometimes in combination with resistant starch and chitosan coatings (Seyedain‐Ardabili et al. 2016). Although polysaccharide‐based encapsulation's biocompatibility and mild processing conditions are advantageous for these systems, multilayer architectures can complicate formulation, increase manufacturing costs, and require additional processing steps. Because of their commercial application and storage benefits, spray drying and freeze drying are also frequently employed for probiotic encapsulation. Heat and dehydration stressors may lower bacterial viability, especially if the protective carriers are not properly chosen (Misra et al. 2021). Emulsification‐based solutions provide an excellent balance between cell protection, scalability, and inclusion in liquid or semisolid food products when compared to these methods (Camelo‐Silva et al. 2022).

W1/O/W2 double emulsions are especially appealing for probiotic administration within emulsification‐based methods since they can enclose bacteria in internal water phases and offer an extra physical barrier to defend against unfavorable environmental conditions. The inclusion of double emulsions in yogurt improved the viability of *L. paracasei* subsp. DC 412 during storage and after simulated gastrointestinal tract exposure, confirming its potential as a probiotic carrier (El Kadri et al. 2018). However, classical double emulsions are prone to destabilization phenomena such as coalescence, creaming, water transfer between phases, and phase separation, which can impact both product consistency and the long‐term protection of probiotics. To overcome these limitations, recent investigations have looked into the use of structuring agents and hydrocolloids in the oil or aqueous phase. Especially, oleogel‐based double emulsions with carnauba wax have recently been shown to enhance the survival of probiotic strains during storage and thermal, freeze–thaw, and simulated gastrointestinal conditions by reinforcing the oil‐phase structure (Varnaitė‐Kapočė et al. 2025). In aqueous phases, several hydrocolloids and stabilizers, such as GG, xanthan gum, pectin, alginate, gelatin, modified starches, and cellulose derivatives, have been used to increase viscosity, reduce droplet mobility, and improve the physical stability of multiple emulsions (Andrade et al. 2018; Muschiolik and Dickinson 2017). However, the specific role of aqueous‐phase biopolymers in optimizing probiotic‐loaded W1/O/W2 emulsions, especially for *L. paracasei* , remains unexplored. The formation of networks of fat crystals in the oil phase could impede the movement of the water droplets and prevent their adhesion (Andrade et al. 2018). Due to its semi‐crystalline, hydrophobic structure, ethyl cellulose (EC), a cellulose derivative, has recently attracted significant attention as an oleogelator (Hosseini et al. 2022). Gelation induction in liquid oil occurs by raising the polymer/oil blend above the EC glass transition temperature (140°C) and subsequently allowing it to cool to room temperature (Davidovich‐Pinhas et al. 2015). Simultaneously, using hydrocolloids such as GG to increase the viscosity of the inner or outer water phases could enhance the kinetic stability of double emulsions and limit the motion of inner droplets. Nevertheless, there is a lack of systematic research on how EC in the oil phase and GG in the aqueous phase work together to adjust the physicochemical characteristics and probiotic delivery efficiency of W1/O/W2 emulsions containing probiotics.

UC is a chronic condition that falls under inflammatory bowel disease (IBD), yet its exact cause remains unknown. The condition is becoming more common worldwide and is marked by the production of harmful free radicals, a lack of antioxidants to neutralize them, and pro‐inflammatory cytokines. Increased reactive oxygen species and lipid peroxidation impair cellular antioxidant mechanisms, leading to colon inflammation (Bernstein et al. 2010). A range of factors, including intestinal health, genetic factors, dietary choices, tobacco use, and daily habits, influence UC. Preserving a stable gut environment is essential for immune function, as any imbalance can trigger more severe colon inflammation. Non‐steroidal anti‐inflammatory drugs (NSAIDs), corticosteroids, and immunosuppressants are some of the medicines used to treat UC. However, these medicines can cause allergies, nausea, and a higher risk of lymphoma, which is why safer alternatives are needed (Dhillon and Singh 2020; Salamatian et al. 2019). UC is associated with reduced levels of *Bifidobacterium* spp. and *Lactobacillus* spp., demonstrating the potential of probiotics in reducing inflammation (Vieira et al. 2023). Research indicates that probiotics may assist in controlling UC by altering the intestinal flora and boosting the immune response of the mucosa (Fuller 1989; Hu et al. 2020). Supplementing with *L. paracasei* helps protect colon tissue from damage, reduce inflammation, and improve gut health. Supplementation alters the diversity of the intestinal microbiota, offering the potential to slow the progression of UC. Limited research has examined the impact of optimizing formulations containing probiotic bacteria on UC treatment. Therefore, this represents a significant knowledge gap at the interface of food‐grade encapsulation science and probiotic‐based UC therapy. In this way, Thakur et al. (2019) developed a spray‐dried mesalamine formulation containing probiotic biomass. This formulation protected animal models from colitis, demonstrating that formulation techniques can enhance the efficacy of therapies. *L. paracasei* CRL 431 has been previously reported as a representative probiotic strain, considering the beneficial intestinal and immunomodulatory properties. Research has demonstrated that it can enhance mucous and systemic immunity, resulting in noticeably elevated levels of vaccine‐specific plasma IgG, IgG1, IgG3, and secretory IgA in saliva (Rizzardini et al. 2012). By altering the gut inflammatory response, administration of *L. casei* CRL 431 has been demonstrated to lessen the severity of *Salmonella enterica* serovar *Typhimurium* pathogenicity in mice (Castillo et al. 2011). Encapsulation of *L. paracasei* CRL 431 in various matrices offers several advantages, primarily focused on enhancing viability and delivery. Thus, the goals of this study included (1) optimizing the content of EC in the oil and GG in the internal and external aqueous phases through application of BBD; (2) assessing the viability, morphology, and rheological properties of optimized double emulsions containing *L. paracasei* CRL 431; and (3) establishing the necessity and efficacy of optimized emulsions in the management of UC in rats.

## 2 Materials and Methods

### 2.1 Culture Preparation

*L. paracasei* (CRL 431) was provided by CHR‐Hansen (Horsholm, Denmark). The microbial culture was prepared by aseptically transferring 0.01 g of the freeze‐dried *L. paracasei* (CRL 431) (Horsholm, Denmark) into 10 mL MRS broth (Merck, Darmstadt, Germany) and incubating for 48 h at 35°C. After that, 1 mL of the subsequent culture was added to MRS broth and incubated under the same conditions. Cells were harvested by centrifugation at 10,000 rpm for 10 min, then washed three times with deionized water. The presence of 10.69 log CFU/mL of the final bacterial concentration was confirmed by OD adjustment to 0.9 by a spectrophotometer (UV‐Vis Array, Photonix Ar 2015) and subsequent culturing on MRS agar (Merck, Darmstadt, Germany) (Mahmoudzadeh et al. 2017).

### 2.2 W1/O/W2 Emulsions Preparation

A 2‐stage method was applied for double emulsion preparation, according to Devanthi et al. (2018), with slight modification. A chemical called EC (18–22 mPa s, 48% ethoxy content, TCI Chemical Co., Tokyo, Japan) was added to sunflower oil in three different amounts (2%, 3%, and 4%). The mixtures were heated at approximately 110°C–120°C under continuous stirring for about 90 min until the mixture became clear, indicating complete dissolution of EC. The mixture was allowed to cool to room temperature and then utilized to prepare the emulsion. To evaluate if the heating treatment for EC dissolution led to any significant lipid oxidation, the oxidative status of the sunflower oil was assessed after the oleogel formation process. The peroxide value of the oil after heating and subsequent cooling was 1.5 meq O^2^/kg oil, suggesting low levels of primary lipid oxidation. The probiotic suspension was added only during the preparation of the inner aqueous phase (W1). For preparation of the initial emulsion (W1/O), polyglycerol polyricinoleate (PGPR; Merck, Darmstadt, Germany; HLB ≈ 4) was added to the cooled oleogel at 5% (w/w of the oil phase). The dispersed phase (W1/O) was prepared by dissolving different concentrations of GG (Merck, Darmstadt, Germany) (0.25%, 0.5%, and 0.75%) and *L. paracasei* harvested cells (9.95 log CFU/mL) in deionized water, then stirring thoroughly with a magnetic stirrer to obtain a uniform solution. A high shear mixer homogenizer (IKA, T25 digital ULTRA TURRAX) was used to make W1/O emulsions at 5800 rpm for 4 min, with a W1: O phase ratio of 30:70. All prepared formulations were stable during 10 days of storage at ambient temperatures. Despite the increased oil phase viscosity due to the oleogelator, the homogenization conditions (5800 rpm) were determined to ensure a balance between emulsion stability and the viability of probiotic cells. This was confirmed by the absence of macroscopic phase separation during storage, as well as the measured droplet size (2400 nm; PDI 0.3). We prepared the outer aqueous phase by dissolving varying amounts of GG (0.25%, 0.5%, and 0.75%) in deionized water and stirring until uniform solutions were obtained. Tween 80 (Merck, Darmstadt, Germany) with HLB of 15 was used in the outer aqueous solution (2%) to stabilize double emulsions. A phase proportion of 60:40 (W1/O: W2) was used to prepare emulsions by homogenization at 8000 rpm for 4 min. The concentration ranges of GG and EC were determined through preliminary trial‐and‐error experiments. When the GG concentration in the inner phase was below 0.25%, the primary emulsion became unstable; thus, 0.25% was determined to be the minimum effective concentration. In contrast, GG concentrations above 0.75% resulted in a sharp increase in viscosity. The EC concentration was optimized based on the extent of oil structuring required to produce an oleogel with appropriate rheological properties and a uniform, stable texture.

### 2.3 Emulsion Stability

The physical stability of the emulsions was evaluated by a centrifugation test after 20 days of storage at room temperature. The samples were subjected to centrifugation (Hettich Universal 320 R, Germany) at 10,000 rpm for a duration of 30 min at a temperature of 25°C ± 2°C. The centrifugation was performed to accelerate phase separation and test the emulsion's resistance to breakdown. After centrifugation, the amount of the emulsified layer remaining in the tube was assessed and contrasted with the initial volume of the emulsion prior to centrifugation. The stability of the emulsion was calculated by the following equation (Atefi et al. 2022):

(1) $$\text{Emulsion stability}:\frac{F_{\mathrm{ev}}}{I_{\mathrm{ev}}}\times 100$$

where *I*_ev_ is the initial total emulsion volume before centrifuging, and *F*_ev_ is the final volume of the emulsified layer remaining after centrifuging.

### 2.4 Droplet Size Analysis

Droplet size distribution of double emulsions was determined after preparing 1:1000 dilutions using a dynamic light scattering (DLS) analyzer (Malvern Instruments, Malvern, UK).

### 2.5 Determination of Encapsulation Efficiency (EE)

The double emulsions that were prepared underwent centrifugation at 4000 rpm for 5 min, resulting in the disruption of the emulsions and the release of all viable cells from the double emulsion system. One mL of liberated aqueous phase was diluted serially with physiological saline (0.9%). The number of living cells was obtained after culturing in MRS agar and incubating at 35°C for 48 h. It was assumed that the centrifugation step breaks the emulsion and permits the recovery of released cells for enumeration, without a correction factor for possible cell adhesion to oil droplets. EE was calculated according to the following equation:

(2) $$\mathrm{EE}=\frac{\text{Number of viable cells in released phase}}{\text{Number of viable cells in initial double emulsion}}\times 100$$

Notably, 10.69 log CFU/mL (5 × 10^10^ CFU/mL) *L. paracasei* dissolved in 10 mL inner aqueous phase, so there was 10.17 log CFU/mL (15 × 10^9^ CFU/mL) in W1/O. With a W1/O: W2 phase ratio set at 60:40, the initial cell count in double emulsions is expected to reach 9.95 log CFU/mL (Pimentel‐González et al. 2009).

### 2.6 Experimental Design

The physicochemical characteristics of the double emulsions were assessed by examining the effects of GG concentration in the inner aqueous phase (*X1*), EC concentration in the oil phase (*X2*), and GG concentration in the outer aqueous phase (*X3*) using a three‐factor, three‐level BBD. The response variables were mean droplet size (*Y1*), emulsion stability (*Y2*), and encapsulation efficiency (*Y3*). The BBD comprised 15 factorial and center‐point combinations. All experimental runs were performed in duplicate to improve measurement reliability, yielding a total of 30 trials. The following second‐order polynomial model modeled the relationship between the independent variables and the responses:

(3) $$Y=\beta_{0}+\sum_{i=1}^{k}\beta_{i}X_{i}+\sum_{i=1}^{k}\beta_{\mathrm{ii}}{X_{i}}^{2}+\sum_{i=1}^{k}\sum_{j=1}^{k}\beta_{\mathrm{ij}}X_{i}X_{j+\varepsilon }$$

Equation 4 was used to assess the model's precision at the suggested optimal point.

(4) $$\text{Relative error}\;\left(\%\right)=\frac{\text{Measured}-\text{Predicted}}{\text{Measured}}\times 100$$

### 2.7 Characterization of Formulations

#### 2.7.1 Microscopy

The morphology of the emulsions was studied by optical microscopy equipped with a digital camera. Images were recorded with a 20× objective lens (magnification ×200). The 20 μm scale bar was shown in the micrographs.

#### 2.7.2 Determination of Acid (AT) and Bile Tolerance (BT)

The tolerance of entrapped and free bacteria to acid and bile conditions was determined (Pimentel‐González et al. 2009). For AT determination, 0.5 mL of double emulsions (containing 9.95 log CFU/mL) or 0.5 mL of free bacteria (in the same bacterial concentration) were dissolved into 9.5 mL of 0.05 M sodium phosphate buffer (Bahar Afshan Co., Tehran, Iran) fixed at pH 2.3 with 0.1 N HCl. Bacterial counts were obtained after incubation at 35°C for 2 h and culturing on MRS agar. To evaluate BT, 0.5 mL of double emulsions (containing 9.95 log CFU/mL) or 0.5 mL of free bacteria (in the same bacterial concentration) were mixed in 9.5 mL MRS broth containing 0.3% w/v bile salts (a 1:1 mixture of sodium cholate and sodium deoxycholate). The solutions were incubated at 35°C for 24 h and then cultured on MRS agar. It is necessary to point out that even though the 24 h incubation period is longer than the usual physiological gastrointestinal transit times (about 3–6 h), this longer exposure was deliberately introduced in order to assess the protective effectiveness of the encapsulation system under more intense bile stress conditions (Mumcu et al. 2025; Veerapagu and Jeya 2017). Furthermore, the same OC formulation was used to create a bacteria‐free (blank) W1/O/W2 emulsion without the inclusion of *L. paracasei* in an attempt to rule out the possibility of microbial contamination. The probiotic‐loaded samples and the blank emulsion were subjected to identical acid (pH 2.3, 2 h, 35°C) and bile (0.3% bile salts, 24 h, 35°C) conditions. Following incubation, serial dilutions were plated on MRS agar and incubated for 48 h at 35°C.

#### 2.7.3 Oscillatory Rheological Measurement

The rheological behavior of the optimized formulations was evaluated after 20 days of storage at room temperature using a Physica MCR 301 rheometer (Anton‐Paar, GmbH, Graz, Austria) with a concentric cylinder geometry measuring system. Oscillatory rheological measurements in frequency sweep mode (0.1–100 1/s) were performed within the linear viscoelastic region at 5% and 1% strain for the HC and OC formulations, respectively.

#### 2.7.4 UC Model in Wistar Rat and Experimental Protocol

The study was performed on male Wistar rats (4–6 weeks old) obtained from the animal care center of Razi Institute (Karaj, Iran). Following 10 days of acclimatization, the animals were kept in controlled conditions with a 12‐h light/dark cycle and unlimited access to water and a normal diet. To induce colitis in fasting rats, a flexible catheter was placed approximately 8 cm rectally while the rodents were sedated. After that, 2 mL of 4% (v/v) acetic acid was gradually added and left for 30 s. The colon was immediately flushed with 2 mL normal saline to remove residual acid, and rats were briefly held in a head‐down position to prevent leakage (Low et al. 2013). To prevent allocation bias, a computer‐generated random number sequence was used to randomly assign the animals to five experimental groups (*n* = 8 per group) following induction. Group 1 (Intact) received oral isotonic saline; Groups 2–5 underwent acetic acid‐induced UC. The groups were treated as follows: Group 2 (Free) received no probiotic; Group 3 (UC + OC) was given optimized W1/O/W2‐encapsulated *L. paracasei* (9.95 log CFU/mL, 1 mL/200 g BW); Group 4 (UC + HC) received the same dose of *L. paracasei* encapsulated in W1/O/W2 emulsions with highest EC and GG concentrations; and Group 5 (UC + Pred) received prednisolone (10 mg/kg/day) via oral gavage.

Throughout the 10‐day experimental period, rats were monitored daily for clinical manifestations of colitis. Using the criteria outlined in Table 1, the Disease Activity Index (DAI), which is the total score (0–3 range) of three medical parameters—body weight loss, stool consistency, and extensive rectal bleeding—was used to measure the severity of the illness. The Kaplan–Meier method was used for survival analysis. The animals were humanely subjected to death at the end of the study, and colon tissues were removed for histopathological evaluation (Bakhtiary et al. 2026). H&E‐stained portions were assessed using a semi‐quantitative scoring method (0–14) to quantify microscopic damage. This index incorporated four key histopathological features: the severity of inflammation, the extent of inflammatory infiltration, crypt damage, and the percentage of tissue involvement. This standardized scoring approach was applied to all experimental groups to provide a comparative quantitative analysis of colonic mucosal injury (Wirtz et al. 2017).

**TABLE 1:** A scoring system for the evaluation of extent and severity of UC.

Score	Weight loss	Stool consistency	Blood feces
0	Negative	Normal	Negative
1	1%–9%	Soft	Red
2	10%–19%	Very soft	Dark red
3	> 20%	Diarrhea	Black

#### 2.7.5 Statistical Analysis

All measurements were executed in triplicate. Statistical evaluation was performed utilizing Minitab software (version 18.0; Minitab Inc., USA). Minitab software was also used to create and analyze the experimental design matrix. One‐way analysis of variance (ANOVA) and the Tukey post hoc test were used to assess significant differences between experimental groups, with a significance level of *p* < 0.05.

## 3 Results and Discussion

### 3.1 Optimization

Table 2 shows droplet size, emulsion stability, and EE of double emulsions as a function of EC and GG concentration in the inner and outer phases. Table 3 details the regression coefficients for the quadratic model of droplet size, emulsion stability, and EE. Different levels of significance were found for each dependent response in the analysis of variance for the model coefficient. The proportion of GG in the inner and outer phases (main effects) significantly influenced droplet size, emulsion stability, and EE (*p* < 0.005). Meanwhile, the EC level (main effects) significantly influenced only droplet size and emulsion stability (*p* < 0.005). All quadratic coefficients were significant for the EE response (*p* < 0.05) (Table 3).

**TABLE 2:** Three‐factor, 3‐level Box–Behnken design for *Lactobacillus paracasei* encapsulation in W1/O/W2 emulsions^a^.

Run	GG in inner phase (%) (*X1*)	EC (%) (*X2*)	GG in outer phase (%) (*X3*)	Average droplet size (nm) (*Y1*)	Emulsion stability (%) (*Y2*)	Encapsulation efficiency (%) (*Y3*)
1	0.25	2	0.5	773.0	60	80.14
2	0.75	2	0.5	1787.0	75	87.18
3	0.25	4	0.5	834.5	65	78.13
4	0.75	4	0.5	2167.0	80	84.89
5	0.25	3	0.25	647.0	50	72.51
6	0.75	3	0.25	1200.5	90	93.68
7	0.25	3	0.75	828.0	90	95.51
8	0.75	3	0.75	2565.0	75	83.89
9	0.5	2	0.25	930.0	55	76.78
10	0.5	4	0.25	999.0	65	75.11
11	0.5	2	0.75	1506	85	90.38
12	0.5	4	0.75	1681.5	90	89.64
13	0.5	3	0.5	1169.6	65	82.91

^a^ *X1* represents the amount of guar gum in the internal phase (W1/O), *X2* represents the amount of ethyl cellulose in oil, and *X3* represents the amount of guar gum in the external phase (W1/O/W2).

**TABLE 3:** Regression coefficients, *R*‐squared, and relative error of predicted quadratic models^a^ ^,^ ^b^ ^,^ ^c^.

Dependent variable	Intercept	*X1*	*X2*	*X3*	*X1* ^*2*^	*X2* ^*2*^	*X3* ^*2*^	*X1X2*	*X1X3*	*X2X3*	*R*‐squared (adj)	Relative error
Average droplet size	2284	−3012	−694	−1513	2008	94.5	228	318.5	4734	106.5	99.21%	5.55%
*p*‐value	0.000	0.000	0.000	0.000	0.000	0.000	0.429	0.000	0.000	0.133	—	—
Emulsion stability	15.6	77.5	−1.88	45.0	60.0	1.25	12.0	0.00	−220.0	−5.0	92.4%	8.90%
*p*‐value	0.000	0.000	0.002	0.000	0.01	0.349	0.000	1.000	0.000	0.33	—	—
Encapsulation efficiency	37.01	50.7	9.08	49.8	27.44	−1.707	33.7	−0.27	−131.2	0.93	92.5%	7.25%
*p*‐value	0.000	0.000	0.078	0.000	0.018	0.019	0.005	0.915	0.000	0.719	—	—

^a^ *X1* represents the amount of guar gum in the internal phase (W1/O), *X2* represents the amount of ethyl cellulose in oil, and *X3* represents the amount of guar gum in the external phase (W1/O/W2).

^b^ *X1* ^2^, *X2* ^2^, and *X3* ^2^ represent the quadratic terms.

^c^ *X1X2*, *X1X3*, and *X2X3* represent interaction terms.

For each response, contour plots are shown in Figure 1. Notably, droplet size decreased when GG concentrations were lowered for both inner and outer phases, and the emulsion became stable when EC and GG concentrations were increased. On the other hand, higher GG concentrations in both the inner and outer phases led to higher EE. The high adjusted R‐squared values (99.21%, 92.4%, and 92.5% for droplet size, emulsion stability, and EE, respectively) and the non‐significance of the lack‐of‐fit test indicate the model's appropriateness. The best results for EE and emulsion stability were found with a GG concentration of 0.25% in the W1 phase and 0.74% in the W2 phase. The smallest droplet size was also found at this concentration. An EC concentration of 2.8% was also optimal for these desired outcomes. Results suggested that gelation of the oil phase by EC and an increase in aqueous‐phase viscosity by GG in the inner and outer phases could increase emulsion stability by 90.5%, EE by 95.8%, and decrease droplet size by 777.5 nm. The results correspond with the observations of Šipailienė et al. (2022), who indicated that the gelation of the W1 phase may enhance the survival rate of *L. plantarum* F1 cells in W1/O/W2 emulsions. The emulsion stability, EE, and droplet size were calculated at the optimal GG and EC concentrations under both measured and predicted conditions to validate the models. The error percentage was then estimated using Equation 4. Error percentage values below 10% indicate the accuracy of the obtained models (Table 3).

**FIGURE 1:**
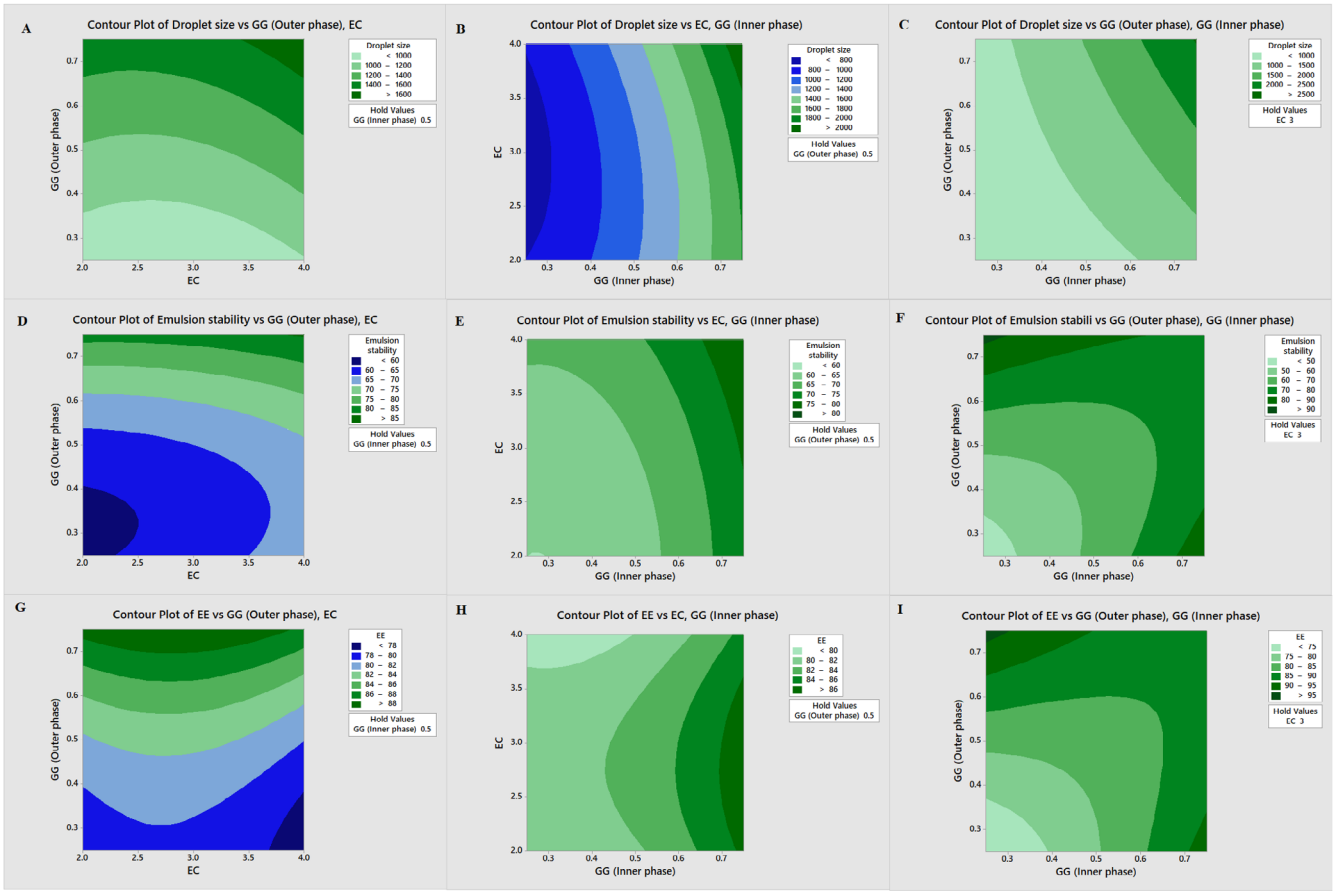
Contour plots for the effect of guar gum (GG) in the inner and outer aqueous phases and ethyl cellulose (EC) in the oil phase. (A–C) droplet size, (D–F) emulsion stability, and (G–I) encapsulation efficiency. Legends indicate the fixed level of the third component in each plot.

### 3.2 Microscopy

Figure 2 illustrates the overall microstructure of double W/O/W emulsions with different formulations. Figure 2a represents W/O/W emulsions synthesized at optimal concentrations (OC), while Figure 2b,c represent emulsions at the highest GG (0.75%) and EC (4%) concentrations (HC) and medium GG (0.5%) and EC (3%) concentrations (MC), respectively. In all formulations, oil droplets are observed in the core of W/O/W emulsions. Double emulsions in OC formulation displayed a one‐droplet‐in‐one‐droplet structure. However, in HC and MC emulsion formulations (Figure 2b,c), in addition to the droplets observed in Figure 2a, large droplets containing numerous dispersed tiny droplets were also observed. This appearance of double emulsions is consistent with findings from previous studies (Matos et al. 2013; Sapei et al. 2012). According to Sapei et al. (2012), gelation of the inner aqueous phase enhances the stability of double emulsions. However, our study indicates that achieving optimized concentrations for average droplet size, EE, and emulsion stability is necessary when the inner and outer aqueous and oil phases are gelled. An increase in GG concentration in both the inner and outer phases leads to larger particles. This may be ascribed to the elevation in the medium's viscosity, which directly influences droplet size (Afoakwa et al. 2008; Román et al. 2018). Although many studies have reported an inverse relationship between viscosity and particle size, some reports also indicate that high viscosity of the aqueous phase can reduce homogenization efficiency by preventing the formation of vortices responsible for droplet dispersion (Amrani et al. 2023). Notably, at the HC formulation, the polydispersity index (PDI) increased to approximately 1, indicating a broader particle size distribution. Therefore, although the average particle size in the experimental design (Table 1) was no more than 2.5 μm, particles with a diameter of 20 μm were also observed due to increased PDI.

**FIGURE 2:**
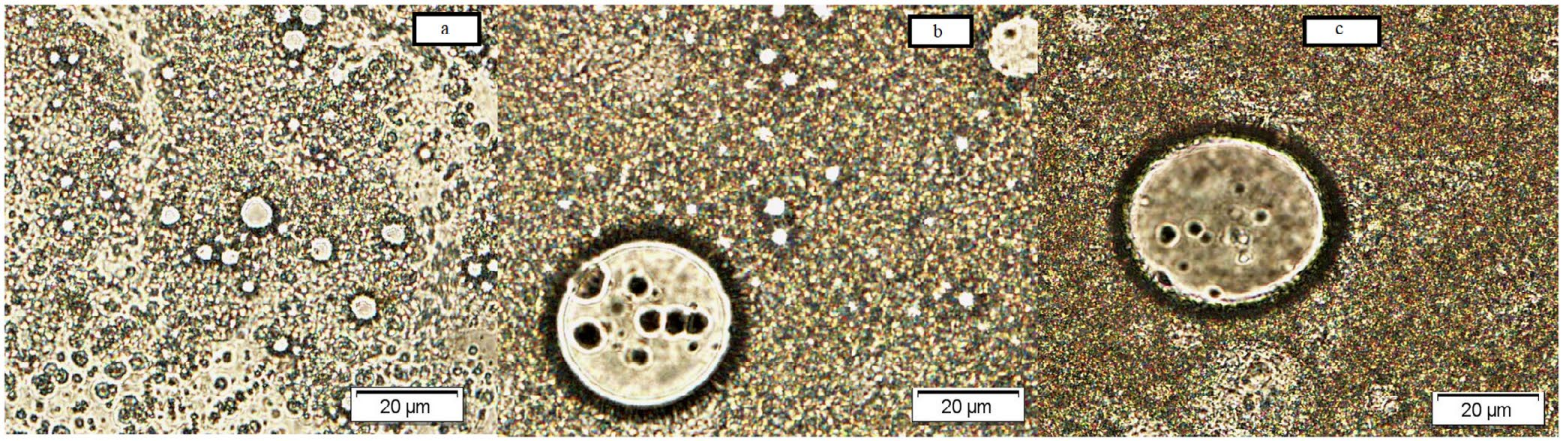
Microscopic images of W1/O/W2 emulsions prepared at different levels of guar gum (GG) and ethyl cellulose (EC): (a) optimized, (b) high, and (c) medium levels. Scale bars = 20 μm.

### 3.3 Determination of AT and BT

Figure 3 shows the survival of free and encapsulated *L. paracasei* in W1/O/W2 emulsions prepared with OC and HC formulations under simulated acid (2 h, 35°C) and bile salt (24 h, 35°C) stresses. The higher physical stability of double emulsions produced at higher GG and EC concentrations (Table 2) was the basis for selecting the most stable formulation, which was compared with the OC formulation.

**FIGURE 3:**
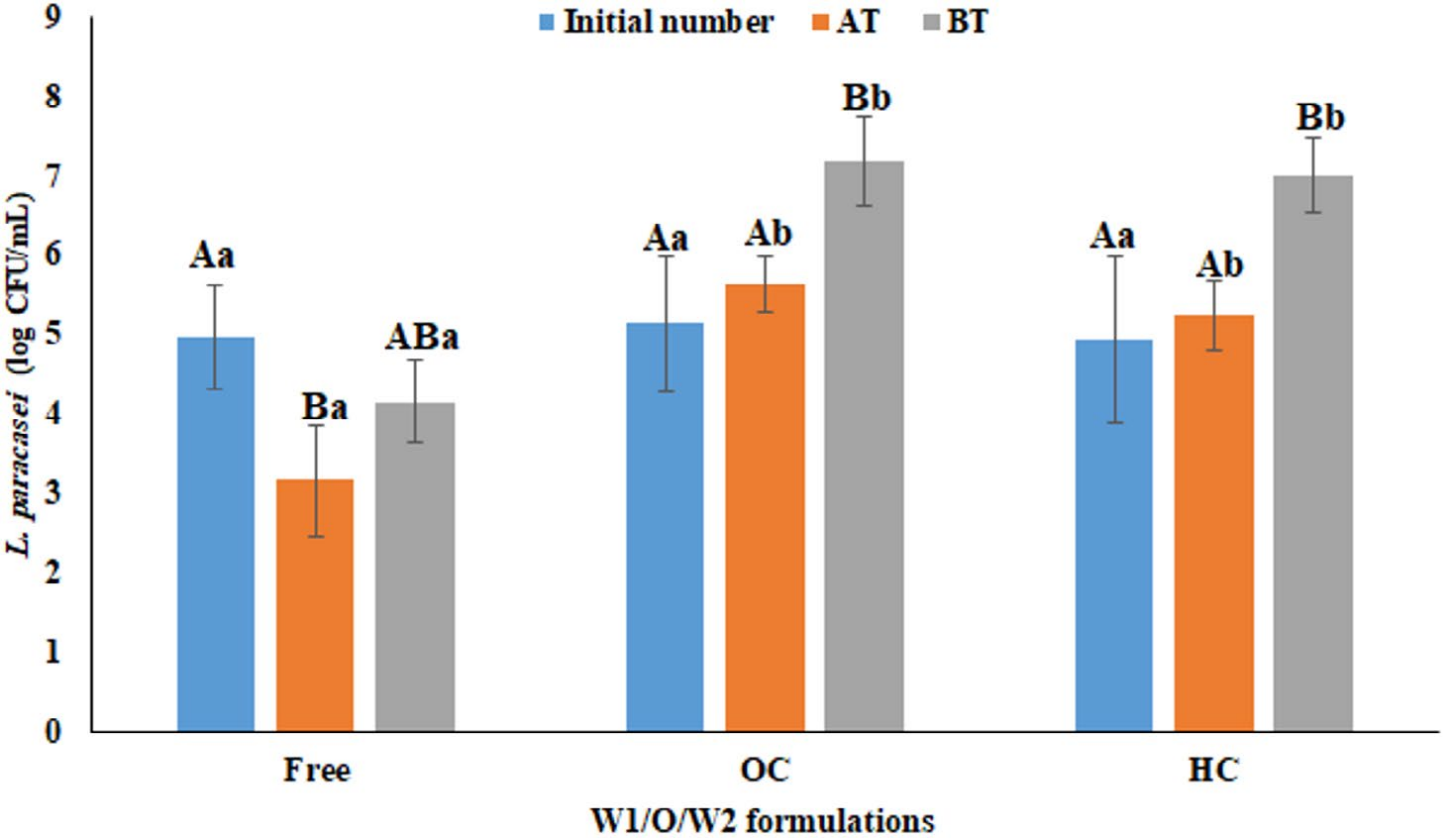
Survival of *L. paracasei* in different W1/O/W2 emulsion formulations (Free, OC, and HC) under varying acid and bile conditions. Different uppercase letters within the same formulation indicate significant differences among treatments (*p* < 0.05), whereas different lowercase letters within the same treatment indicate significant differences among formulations (*p* < 0.05).

No significant difference was observed between the initial viable cell count of free and freshly encapsulated cells (*p* > 0.05). Under acidic conditions, free *L. paracasei* showed the lowest viable cell count (3.18 log CFU/mL), confirming the sensitivity of non‐encapsulated cells to low pH. In contrast, encapsulated *L. paracasei* maintained a significantly higher viable cell count in both formulations (*p* < 0.05), indicating that encapsulation reduced the acid‐induced loss of viability (Solanki et al. 2013; Šušković et al. 2000).

The recoverable viable counts of *L. paracasei* in the OC W1/O/W2 formulation were increased from 5.15 to 7.20 log CFU/mL after 24 h of bile salt exposure (*p* < 0.05). Under identical experimental conditions, no colonies were seen when 0.1 mL of the 10^−1^ dilution was plated on MRS agar, indicating a detection limit of 100 CFU/mL (2 log CFU/mL) for the bacteria‐free emulsion control (Supplementary 1). This finding suggests that microbial contamination of the emulsion was unlikely to account for the increased recoverable viable counts (Supplementary 1). Since the bile tolerance assay was conducted in MRS broth containing 0.3% bile salts at 35°C for 24 h, bacterial proliferation during the exposure period cannot be excluded, as MRS broth provides nutrients that may support bacterial growth despite the presence of bile salts. In this way, Ahn et al. (2002) observed that *L. acidophilus* isolates were able to grow in MRS broth containing 0.3% (w/v) bile, indicating the isolates' ability to survive and grow in the intestinal tract. Furthermore, a more thorough recovery in the plate enumeration could be explained by the bile exposure and subsequent sample processing, which encouraged the release and dispersal of previously trapped cells. This is in line with earlier results of probiotic double‐emulsion systems that showed apparent survival values higher than 100% following exposure to gastrointestinal simulations. For instance, 128% of *L. rhamnosus* trapped in a W1/O/W2 double emulsion survived bile exposure, according to Pimentel‐González et al. (2009). Repairing sublethally damaged cells is another reason. Probiotic bacteria can adapt to bile stress, and bile salts have detergent‐like effects on bacterial cell membranes and membrane‐associated components (Singh et al. 2022). Therefore, upon subculturing on MRS agar, a fraction of the physiologically damaged yet recoverable cells may undergo resuscitation and become culturable again. Furthermore, the possible role of GG should be assessed with caution. Partially hydrolyzed GG has been shown to support the growth of a number of lactic acid bacteria and has shown potential prebiotic activity (Mudgil et al. 2018); however, the mentioned study used partially hydrolyzed GG, whereas the present formulation contained native GG. Thus, it is not possible to determine the exact contribution of bacterial proliferation, cell release, and recovery in the present bile‐stress assay.

Overall, exposure to acid or bile did not reduce the viability of encapsulated bacteria in any of the formulations (*p* > 0.05), and survival tended to be higher in the OC formulation. Adverse conditions in the gastrointestinal tract, including gastric acid and bile salts, can markedly reduce the viability of probiotics and compromise the structural integrity of some hydrogels (Li et al. 2023). Oleogels based on EC establish a non‐crystalline, thermomechanically stable network that exhibits reduced susceptibility to melt/recrystallization transitions relative to comparable methods (Davidovich‐Pinhas et al. 2016). This property can be beneficial for probiotic protection, especially in double emulsions, where structural integrity is crucial for the controlled release and protection of bioactive compounds (García‐Ortega et al. 2021). Wax‐based oleogels are mainly stabilized by the crystallization of fat crystals that form a 3D crystalline network. These systems typically exhibit solid‐fat characteristics and may have elevated melting points. Wax oleogels can alter the polymorphic transitions of oils and facilitate the production of solid crystals, thus enhancing storage stability, particularly under fluctuating temperature settings (Fu et al. 2025; Gu et al. 2023). Alternative gelation techniques frequently employed in probiotic distribution encompass protein or polysaccharide hydrogel networks (e.g., alginate, whey protein, and pectin), primarily safeguarding cells by creating hydrated matrices that mitigate pH fluctuations and enzymatic degradation (Guo et al. 2026; Kwiecień and Kwiecień 2018). The results obtained collectively suggest that the structural integrity of W1/O/W2 emulsions can be enhanced by optimizing stabilizer concentrations (GG and EC) and improving probiotic survival and recovery under gastrointestinal‐like stresses, indicating their potential use in functional foods and dietary supplement delivery systems (Shi et al. 2024).

### 3.4 Oscillatory Rheological Measurement

Figure 4 illustrates the variations in complex viscosity (η*), storage modulus (G′), and loss modulus (G″) as a function of frequency for the double emulsions (OC and HC). In both formulations, η* diminished with rising angular frequency, signifying shear‐thinning behavior, typically found in emulsions stabilized by hydrocolloids like GG and locust bean gum, which can significantly affect viscoelastic characteristics (Ercelebi and Ibanoglu 2010; Supplementary 2). At lower angular frequencies, the HC emulsion had higher η* than the OC emulsion; nevertheless, the curves intersected in the intermediate frequency region (around 2.5–4 rad/s), showing that the complex viscosity of the two formulations was relative and dependent on frequency.

**FIGURE 4:**
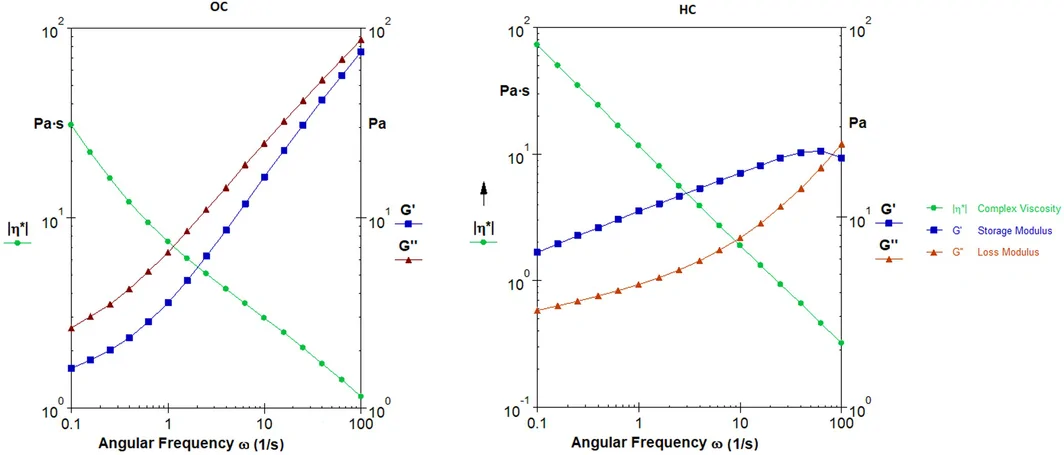
Frequency‐dependent rheological properties of W1/O/W2 emulsions prepared with optimized (OC) and high (HC) levels of guar gum and ethyl cellulose.

In OC double emulsions, both G′ and G″ increased with frequency. At the same time, G″ remained higher than G′ throughout the tested range, indicating predominantly viscous behavior, which is typical of emulsions containing lower concentrations of stabilizing agents (Zhu et al. 2020). However, the gap between G′ and G″ decreased at higher frequencies, suggesting that the structure may exhibit slightly greater elasticity under rapid deformation. For HC double emulsions, G′ and G″ also increased with frequency. However, G′ exceeded G″ over much of the frequency range, particularly at intermediate frequencies, indicating a more gel‐like or solid‐like structure likely associated with the higher concentrations of GG and EC (Lorenzo et al. 2011).

Higher values of G′ may indicate a more structured viscoelastic network. A more structured network could improve physical stability, but at the same time, could negatively affect mass transfer and the spatial distribution of the encapsulated components (Patel et al. 2015). A very dense network with lower internal mobility and more compact internal structure may limit structural rearrangements and diffusion processes during gastrointestinal transit (Caldero et al. 2012). Since the bacteria were trapped in the inner aqueous phase (W1), the relevant diffusion processes would involve either the penetration of gastrointestinal fluids into W1 or the release of bacteria from W1 into the external environment during digestion. Therefore, an overly rigid system, while protective, may limit the controlled release at the site of action. The OC formulation exhibited relatively lower viscosity and more fluid‐like characteristics. These properties could, in principle, provide a less restrictive internal environment, potentially allowing the dispersed phases to rearrange more readily. Such behavior has been proposed in previous studies as a factor that may facilitate a more uniform distribution of microorganisms within the inner aqueous phase (Mehrnia et al. 2017). However, oscillatory rheological measurements were only performed after 20 days of storage. Consequently, although these measurements characterize the viscoelastic behavior of the emulsions at the end of the storage period, the absence of Day 1 measurements limits the strength of the suggested relationship between rheological properties and storage stability and makes it impossible to directly evaluate rheological changes during storage.

### 3.5 Treatment of UC

The encapsulation of *L. paracasei* in double emulsion systems has markedly (*p* < 0.05) enhanced survival rates and diminished death in rats afflicted with colitis. The survival rate of rats given *L. paracasei* in W1/O/W2 emulsion systems stabilized with OC (UC + OC) was higher than that of rats given the immunosuppressant prednisolone (*p* > 0.05) (Figure 5b). Furthermore, the Disease Activity Index (DAI), which assesses the severity of symptoms, was lowest in the OC formulation group and showed no significant difference from the prednisolone group (*p* > 0.05). A statistically significant difference (*p* < 0.05) was noted in comparison to the HC probiotic formulation group (UC + HC). The Free group, which did not receive therapy, exhibited the highest DAI ratings (*p* < 0.05), showing that the encapsulated probiotic significantly alleviated colitis symptoms (Figure 5c). Preliminary tests were conducted to evaluate the possible anti‐inflammatory effect of the blank emulsion (the carrier devoid of *L. paracasei* ). The macroscopic and histological ratings in the blank carrier group were not substantially different from those in the Free group (Supplementary 3).

**FIGURE 5:**
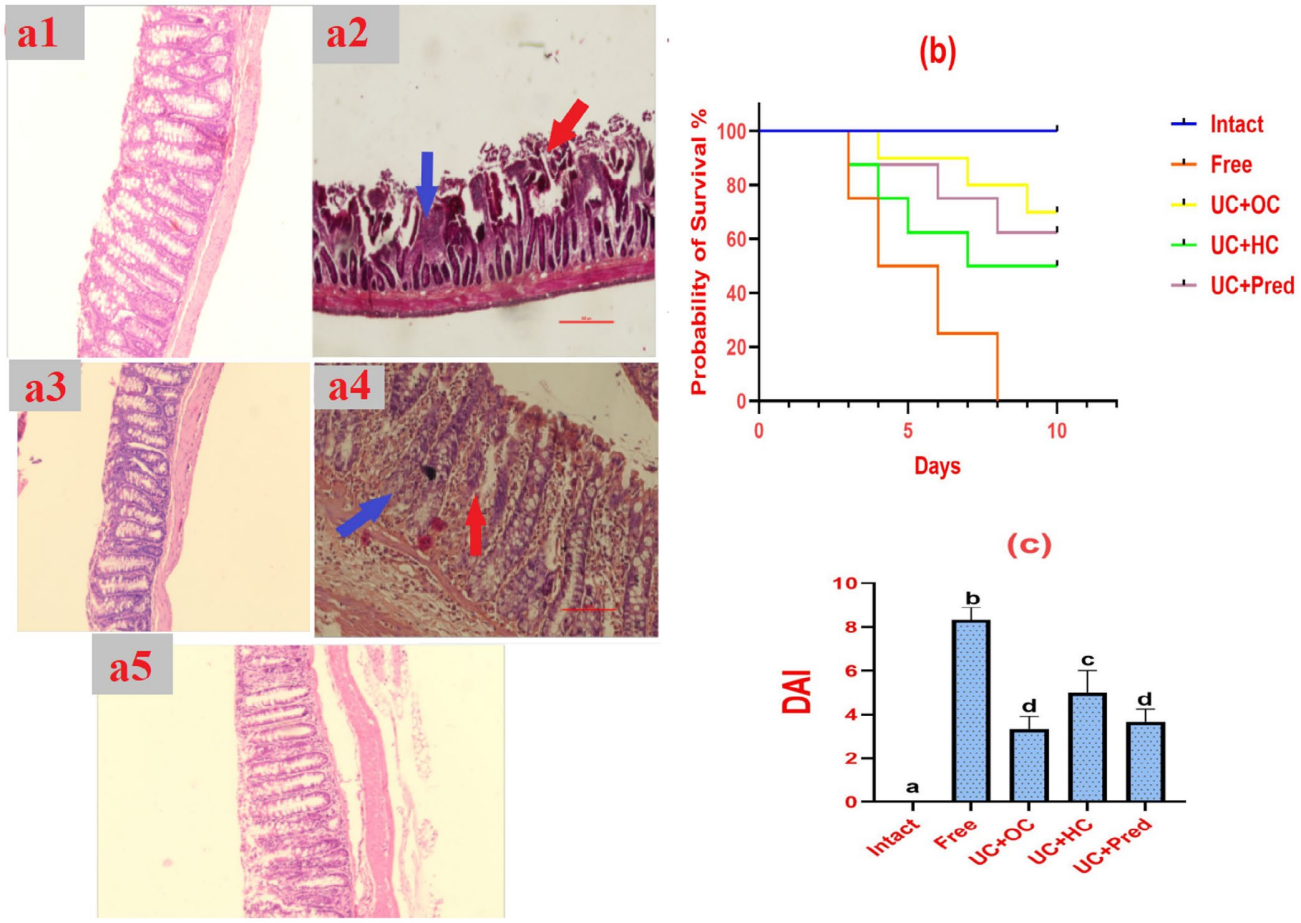
Pathological sections (a1–a5), survival rate (b), and disease activity index (c) in rats with UC after administration of *L. paracasei* encapsulated in W1/O/W2 emulsions (OC or HC) or prednisolone. (a1) Intact group; (a2) UC‐induced untreated group; (a3) UC + OC; (a4) UC + HC; (a5) UC + prednisolone. H&E staining, 100×. Statistical significance was considered at *p* < 0.05.

The pathology tests show that the intestinal crypts and mucosa are healthy and uniform in the intact group, with no signs of damage (Figure 5a1). In contrast, the Free group showed extensive mucosal injury, including detached crypts, epithelial disruption, inflammatory infiltration, and necrosis (Figure 5a2). Mucosal structure was partially restored in the UC + HC group, but inflammatory infiltration and local necrosis persisted (Figure 5a3). The OC formulation receiving animals (UC + OC) showed a significant improvement in mucosal integrity and a reduction in inflammatory indicators, leading to tissue profiles similar to those of the prednisolone group (Figure 5a4,a5). The semi‐quantitative histopathology ratings (Supplementary 3) were in agreement with the microscopic observations. The Free group had the highest scores of tissue damage, demonstrating substantial colonic damage, whereas the intact group had minor histological changes. The UC + OC group showed a significant reduction (*p* < 0.05) in the histopathological score among the treated groups, reflecting the preservation of mucosal architecture and reduction of inflammation similar to the prednisolone‐treated group (Supplementary 4). These results suggest the potential of *L. paracasei* administered in optimized double‐emulsion systems to have anti‐inflammatory and gut‐modulating effects (Zeng et al. 2021). Previous studies have also reported that the structural properties of polysaccharide‐based encapsulation systems greatly affect the release behavior of encapsulated microorganisms under gastrointestinal conditions. Specifically, chitosan‐coated alginate systems have been proven to give a more regulated and progressive release profile in simulated intestinal environments and to protect encapsulated bacteria from gastrointestinal conditions. The time course would facilitate release control in the small intestine but not extend to the distal parts of the large colon, which is a goal for UC medication (Cook et al. 2012). For colonic delivery, such as in UC management, an optimal balance between retention and timely release is critical. On the one hand, an excessively rigid matrix may delay release beyond the desired intestinal region, whereas a system with moderate viscoelastic properties may allow more appropriate release kinetics (Caldero et al. 2012). However, parameters such as release kinetics, mucoadhesion, gastrointestinal transit behavior, and bacterial colonization were not directly assessed in the present study. Therefore, any proposed association between the rheological properties of the formulations and the improved in vivo performance of the OC system must be evaluated with caution and viewed as a plausible explanation rather than a validated mechanistic effect. The efficacy of *L. paracasei* in different formulations, including various dietary supplements, needs further investigation, as the survival and activity of probiotic strains are strongly dependent on the precise delivery vehicle. However, the long‐term clinical potential of this probiotic delivery system remains to be established by translational research on different dosages and the impact of the specific delivery platforms on microbial viability and therapeutic outcome (Dang et al. 2018).

The acetic acid‐induced colitis model used in this study provides a robust and effective way to evaluate acute inflammatory responses and the protective effects of our novel encapsulation system. However, it does not fully reproduce the complex, chronic, and relapsing nature of human UC (Wirtz et al. 2017). The acetic acid model primarily mimics acute mucosal injury rather than the chronic immune‐mediated disease observed in human patients. Therefore, our results highlight the immediate protective and anti‐inflammatory potential of the optimized *L. paracasei* ‐loaded W1/O/W2 emulsions. To close this gap and translate these delivery systems into clinical application, future research should focus on confirming their efficacy in models that better resemble chronic conditions, for example, Dextran Sodium Sulfate (DSS)‐induced colitis or genetically altered mice (Bahrami et al. 2022).

## 4 Limitations

There are several limitations of the present study that should be noted. First, the acetic acid‐induced colitis model mainly recapitulates acute mucosal injury and does not fully recapitulate the chronic, immune‐mediated, relapsing nature of human UC. Therefore, although our results show the short‐term protective and anti‐inflammatory potential of the optimized W1/O/W2 emulsions, the extrapolation to clinical UC should be cautiously done and validated in chronic models (e.g., DSS‐induced colitis). Second, release kinetics, mucoadhesion, gastrointestinal transit, and bacterial colonization were not directly measured, and the proposed correlation of the more fluid‐like rheological properties of the OC formulation with its superior in vivo efficacy is a plausible but unproven mechanistic explanation. Third, the rheological characterization was carried out after 20 days of storage, so time‐dependent changes in viscoelasticity could not be evaluated. While these limitations do not negate the observed advantages of the OC formulation under the experimental conditions used, they suggest that conclusions related to long‐term stability, accurate delivery mechanisms, and clinical translation should be considered tentative, and further investigation is needed.

## 5 Conclusion

In this study, we optimized water‐in‐oil‐in‐water (W1/O/W2) double emulsion systems for the encapsulation of *L. paracasei* . The results demonstrated a stable, effective delivery system with potential health benefits for the management of UC. The OC formulation maintained the viability of *L. paracasei* in gastrointestinal‐like conditions, while no significant difference in viability was seen between the OC and HC formulations. It demonstrated notable anti‐inflammatory effects in a Wistar rat model of UC, resulting in non‐significantly lower DAI scores and mucosal healing comparable to that observed with prednisolone. The better performance of this formulation could be due to the rheological properties of the OC emulsion, which showed the best compromise between viscosity and elasticity. The positive in vivo results obtained with the OC formulation could be related to its rheological properties, which ensured an optimal compromise between viscosity and elasticity. It can be hypothesized that these features favor the physical stability of the emulsion and the distribution of viable cells, thus possibly supporting probiotic delivery under gastrointestinal conditions.

## Author Contributions

**Leila Mahmoudzadeh:** methodology, investigation, resources, writing – original draft, writing – review and editing. **Sara Chadorshabi:** methodology, investigation. **Ehsan Shamloo:** investigation, writing – review and editing. **Mahnaz Tabibiazar:** methodology, writing – review and editing. **Aziz Homayouni‐Rad:** methodology, validation. **Maryam Mahmoudzadeh:** conceptualization, supervision, investigation, validation, funding acquisition, writing – review and editing. **Hashem Andishmand:** supervision, resources, writing – review and editing.

## Funding

This research was financially supported by the vice‐chancellor of research of Tabriz University of Medical Sciences, Iran (Grant number: 63775).

## Ethics Statement

All animal experiments were approved by the Research Ethics Committee of Tabriz University of Medical Sciences (Ethical code: IR.TBZMED.VCR.REC.1398.206). All animal procedures were performed in accordance with institutional guidelines and national regulations for the care and use of laboratory animals. All possible steps were taken to minimize animal suffering and to reduce the number of animals used.

## Conflicts of Interest

The authors declare no conflicts of interest.

## Supporting information


**Supplementary 1.** Representative images of MRS agar plates showing the survival of *Lactobacillus paracasei* in different dilutions. (A) Growth of *L. paracasei* released from the OC formulation after being subjected to acid and bile salt stress. (B) Negative control (OC formulation devoid of *L. paracasei* ).

**Supplementary 2.** Raw rheological measurement datasets (viscosity, storage modulus G′, and loss modulus G′′) for OC and HC formulations, recorded using an Anton Paar rheometer.

**Supplementary 3.** Pathological sections in rats with UC after administration of blank W1/O/W2 emulsions.

**Supplementary 4.** Effects of different treatments on semi‐quantitative histopathology ratings in an acetic acid‐induced colitis rat model. Group 1: Intact; Group 2: UC control (no treatment); Group 3: UC + OC (optimized W1/O/W2‐encapsulated *L. paracasei* ); Group 4: UC + HC (high EC/GG concentration encapsulated *L. paracasei* ); Group 5: UC + Pred (prednisolone). Data are presented as mean ± SD.

## Data Availability

The data that support the findings of this study are available from the corresponding author upon reasonable request.
